# Impact of Proprioceptive Neuromuscular Facilitation Technique for Early Rehabilitation to Restore Motor Impairments in a Classic Case of Left Middle Cerebral Artery Stroke

**DOI:** 10.7759/cureus.31222

**Published:** 2022-11-07

**Authors:** Fatimah Kazi, Ragini Dadgal, Vikrant G Salphale

**Affiliations:** 1 Department of Neurophysiotherapy, Ravi Nair Physiotherapy College, Datta Meghe Institute of Medical Sciences, Wardha, IND

**Keywords:** motor impairement, stroke rehabilitation, physiotherapy, proprioceptive neuromuscular facilitation, middle cerebral artery

## Abstract

An abrupt hemorrhage or ischemia causes acute onset of stroke. The characteristic feature of hemiplegia is the loss of voluntary movement with the alteration of muscle tone, reflexes, and sensation. In this case, we present a 56-year-old man who suffered from right hemiplegia, facial palsy, and expressive aphasia. The MRI of the brain revealed a hemorrhagic transformation of acute infarct in the left frontoparietal-temporo-occipital lobe. The patient was managed immediately by medical interventions. The physiotherapy treatment was initiated after the stabilization of acute symptoms at an early stage. This case report details the management of the patient with physical therapy and highlights the advantages of exercise therapy, particularly the proprioceptive neuromuscular facilitation technique for enhancing the patient's condition by incorporating physiotherapy protocol from an early stage.

## Introduction

A cerebrovascular accident, more commonly known as a stroke, is a fatal condition that requires emergency and high-priority medical treatment. It can lead to long-term disabilities and even death in severe cases [1]. This cerebrovascular event happens whenever the blood flow to the brain is hampered (known as ischemic stroke), or sudden bleeding from a blood vessel occurs in the brain (known as hemorrhagic stroke). A clot or an infarct prevents blood flow. This clot in areas of severely reduced blood flow causes excitotoxic and necrotic cell death within minutes, and tissue undergoes irreversible damage in the absence of reperfusion [2]. This leads to paralysis or paresis of the affected side of the body, reduced sensation, abnormalities of tone and reflexes, disruption in postural control and balance, apraxia, unilateral neglect, and anosognosia. It is generally observed that the artery affected by stroke is the middle cerebral artery (MCA) [3]. The MCA is a critical artery that supplies the frontal, temporal, and parietal lobes as well as subcortical regions such as the internal capsule, corona radiata, the outer portion of the globus pallidus, and the caudate nucleus. The hallmarks of the MCA occlusion stroke are contralateral spastic hemiplegia and loss of sensation in the face and upper extremities [4]. The face and the upper extremity are more involved than the lower limb. Involvement of the language centers of the dominant hemispheres of the brain can lead to aphasia [5]. Usually, CT scan and MRI of brain are carried out to confirm the diagnosis of stroke. Medical intervention usually revolves around drug therapy, thrombolysis, thrombectomy, administering anticoagulant and antiplatelet medications, and surgical intervention.

A person experiences severe to moderate impairments in their motor skills and other disabilities following a stroke [6]. These disabilities can be very limiting for a person to carry out activities in daily life. Physiotherapy aims to help the patient achieve greater independence and improve functional capacity, including gait, posture, strength, transfers, coordination, and balance. Proprioceptive neuromuscular facilitation (PNF) is a rehabilitation method developed by Dr. Herman Kabat and Miss Margaret Knott. The philosophy of this technique is based upon the stimulation of the proprioceptors to obtain a maximal response from the neuromuscular system. PNF has been used in treating various neurological and musculoskeletal conditions. It is a successful method for treating motor impairments and improving gait parameters after an acute stroke [7]. The efficiency of physical therapy after a stroke is relatively high [8]. This case study presents a 56-year-old man, exhibiting classic clinical features of a middle cerebral artery stroke. He was managed medically and underwent physiotherapy treatment at an early stage, which helped him to recover from motor impairments and improve his quality of life.

## Case presentation

A 56-year-old man, a steel factory worker with right-hand dominance, came in a wheelchair driven by his wife to the physiotherapy outpatient department. On initial assessment, the patient presented with right-sided weakness of the upper limb, lower limb, and right side of the face with more involvement of the upper limb. The patient was not able to communicate because of aphasia. He had difficulty maintaining balance, inability to use his right hand, and had difficulty performing activities of daily living. The wife reported the patient's history as follows: the patient was apparently alright when he experienced a common cold and had a fever one fine day. The patient consulted a doctor in a private hospital nearby and started taking medications for the same. He felt alright, and the fever reduced slightly after taking the medicines. After two days he suffered from a paralytic attack. The patient's wife complained that the first symptom she noticed was that he was unable to speak, and there was a sudden weakness in his arm and leg, causing the patient to fall off his chair. He was then brought to the emergency unit of Acharya Vinoba Bhave Rural Hospital, Wardha, India; immediate medical management was initiated; then he was admitted to the inpatient department. The patient was investigated for the cause of hemiplegia with an initial CT scan followed by an MRI to confirm the diagnosis. The patient was initially managed with medical treatment including physiotherapy for a period of one month. After the patient became stable, he was discharged and referred to the physiotherapy outpatient department for further rehabilitation.

Clinical findings

**Figure 1:**
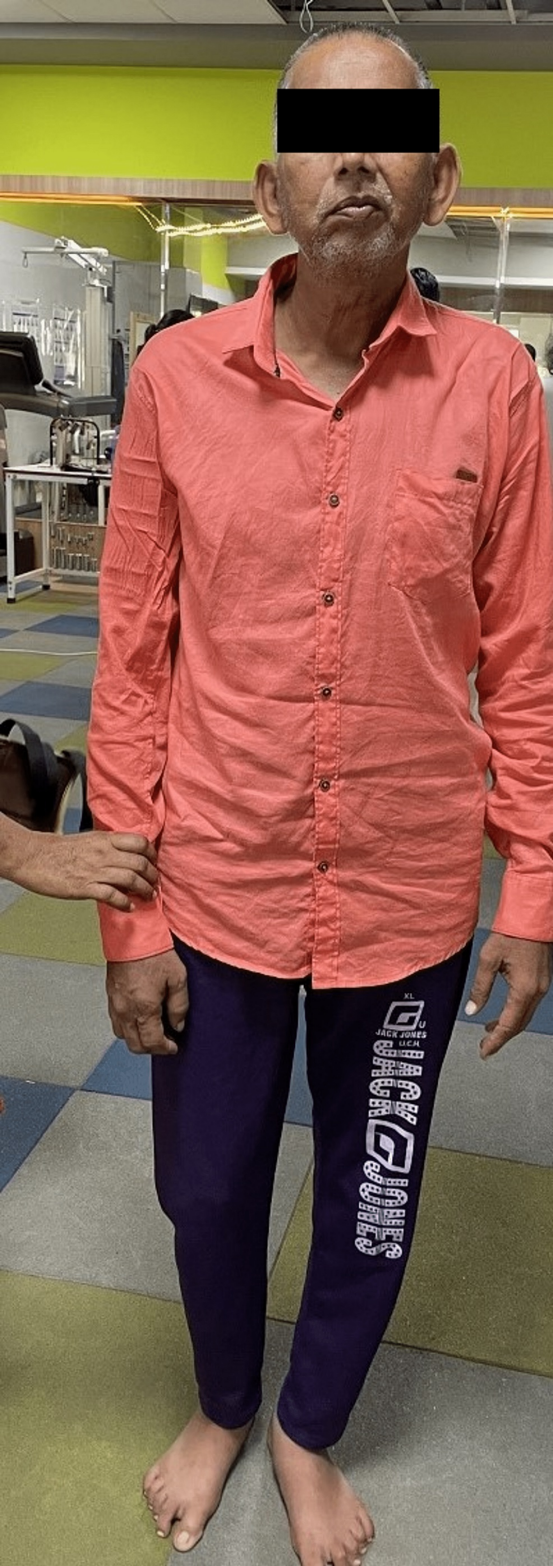
Patient stands with assistance (anterior view)

**Figure 2:**
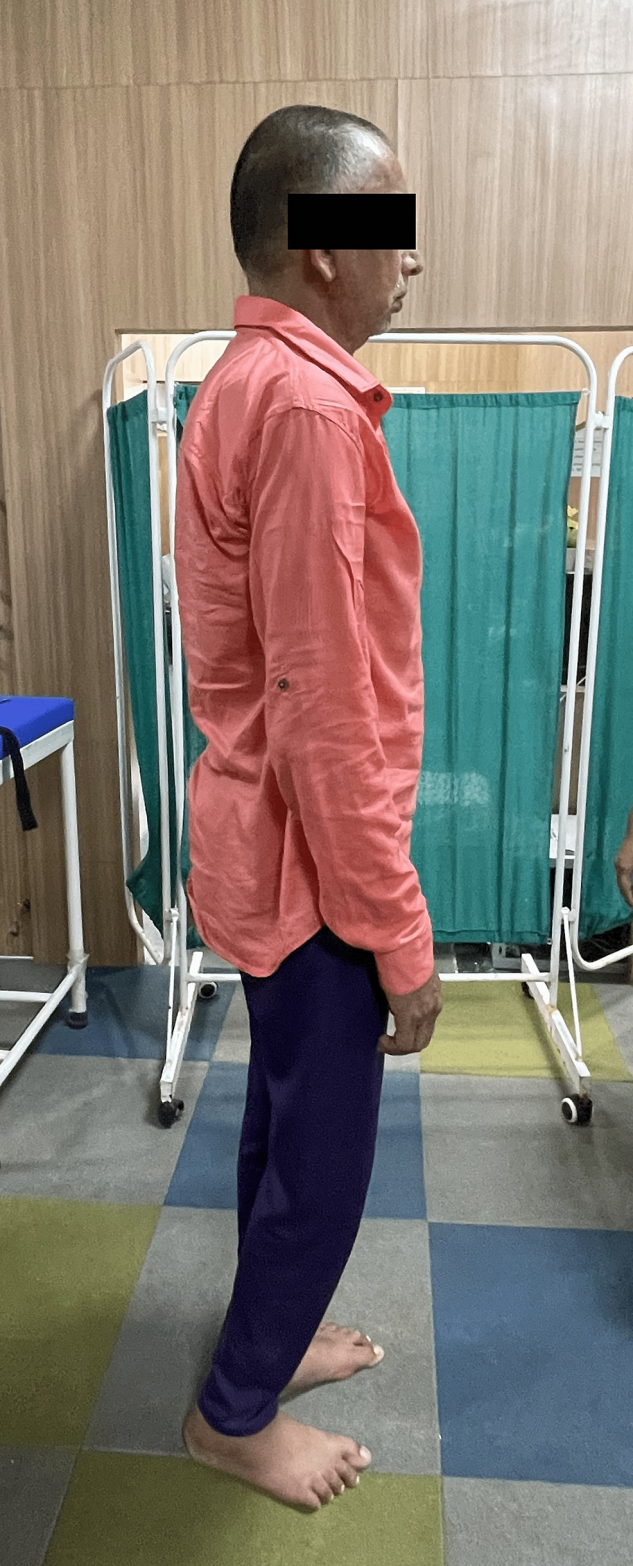
Patient stands (sagittal view)

The patient was conscious and oriented to time, place, and person. On observation, the right shoulder was depressed, and in adduction, internal rotation and the elbow was flexed and the forearm was pronated, as depicted in Figure 1. While viewing from the right sagittal plane, as shown in Figure 2, the patient appeared to have pelvic retraction and elevation, hip adduction, and knee flexion. His vision was good, his hearing was intact, and he had no short-term or long-term memory loss. The patient suffered from expressive aphasia. Tendon jerks tested on the first day are mentioned in Table 1. Tone examination using the modified Ashworth scale on the first day of assessment is depicted in Table 2. Outcome measures like the Motor Assessment Scale, Dynamic Gait Index, and Sunnybrook Facial Grading System assessed on the first day are described in Table 3.

**Table 1:** Deep tendon reflexes

DEEP TENDON REFLEXES		
REFLEX	RIGHT	LEFT
Biceps	3+	2+
Triceps	3+	2+
Supinator	3+	2+
Knee	3+	2+
Ankle	3+	2+

**Table 2:** Tone examination using MAS

Modified Ashworth Scale (MAS)		
	Right	Left
Shoulder flexors	1+	Normal
Elbow flexors	1+	Normal
Wrist flexors and extensors	1+	Normal
Hip flexors	1	Normal
Knee flexors and extensors	1	Normal
Ankle flexors and extensors	1	Normal

**Table 3:** Pre-treatment score of the outcome measures, such as the Motor Assessment Scale, Dynamic Gait Index, and Sunnybrook Facial Grading System

	Pre-Treatment Score	
	Score obtained	Maximum score
Motor assessment scale	24	54
The dynamic Gait Index score	12	24
Sunnybrook Facial Grading System	58	100

Clinical diagnosis

MRI brain was performed on the same day. It revealed altered signal intensity in the left frontoparietal-temporo-occipital lobe appearing isointense on T1-weighted image (T1WI), hyperintense on T2-weighted/fluid-attenuated inversion recovery sequence (T2W/FLAIR) and low signal intensity on apparent diffusion coefficient (ADC).

Physiotherapeutic interventions

**Figure 3:**
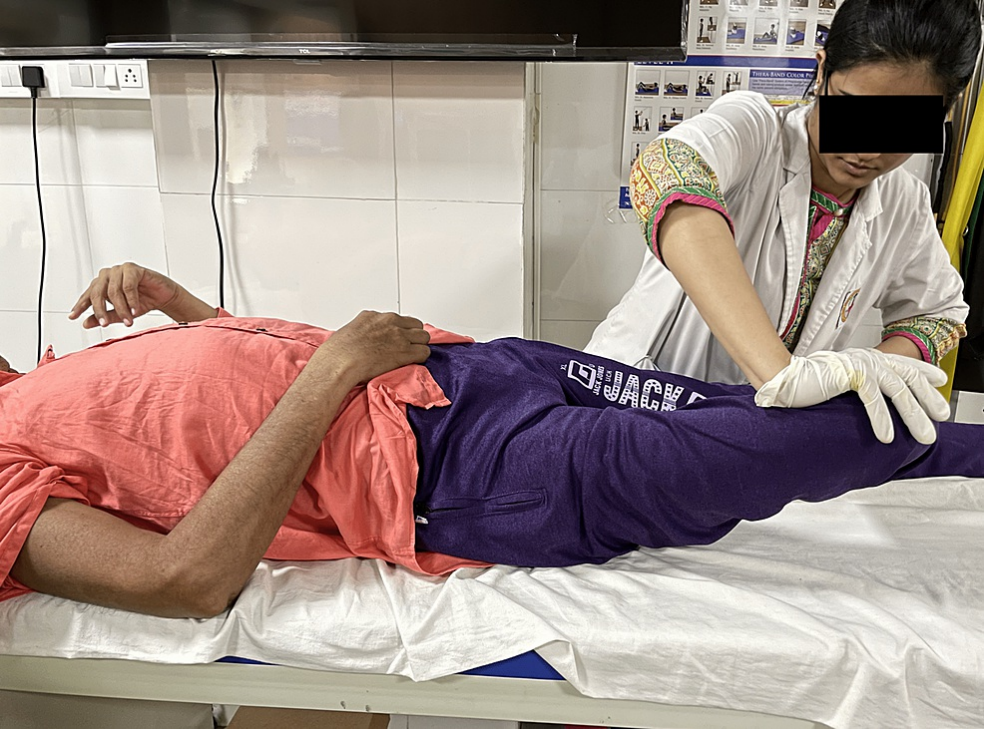
Represents adductor stretching

**Figure 4:**
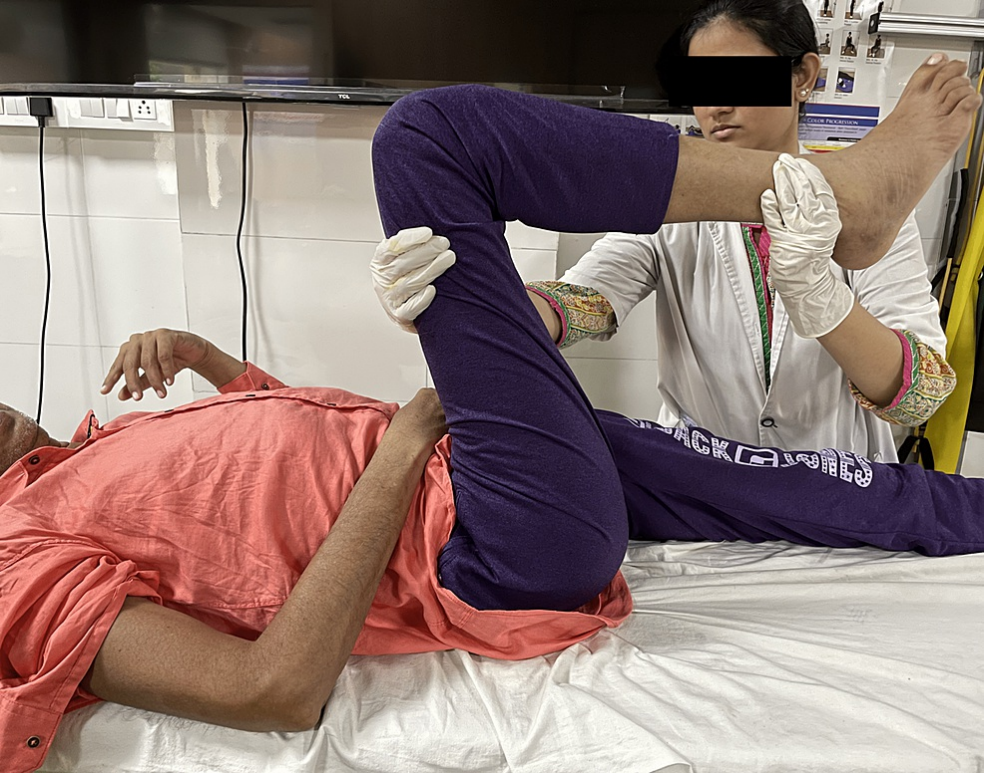
Represents PNF pattern for lower limb PNF = proprioceptive neuromuscular facilitation

**Figure 5:**
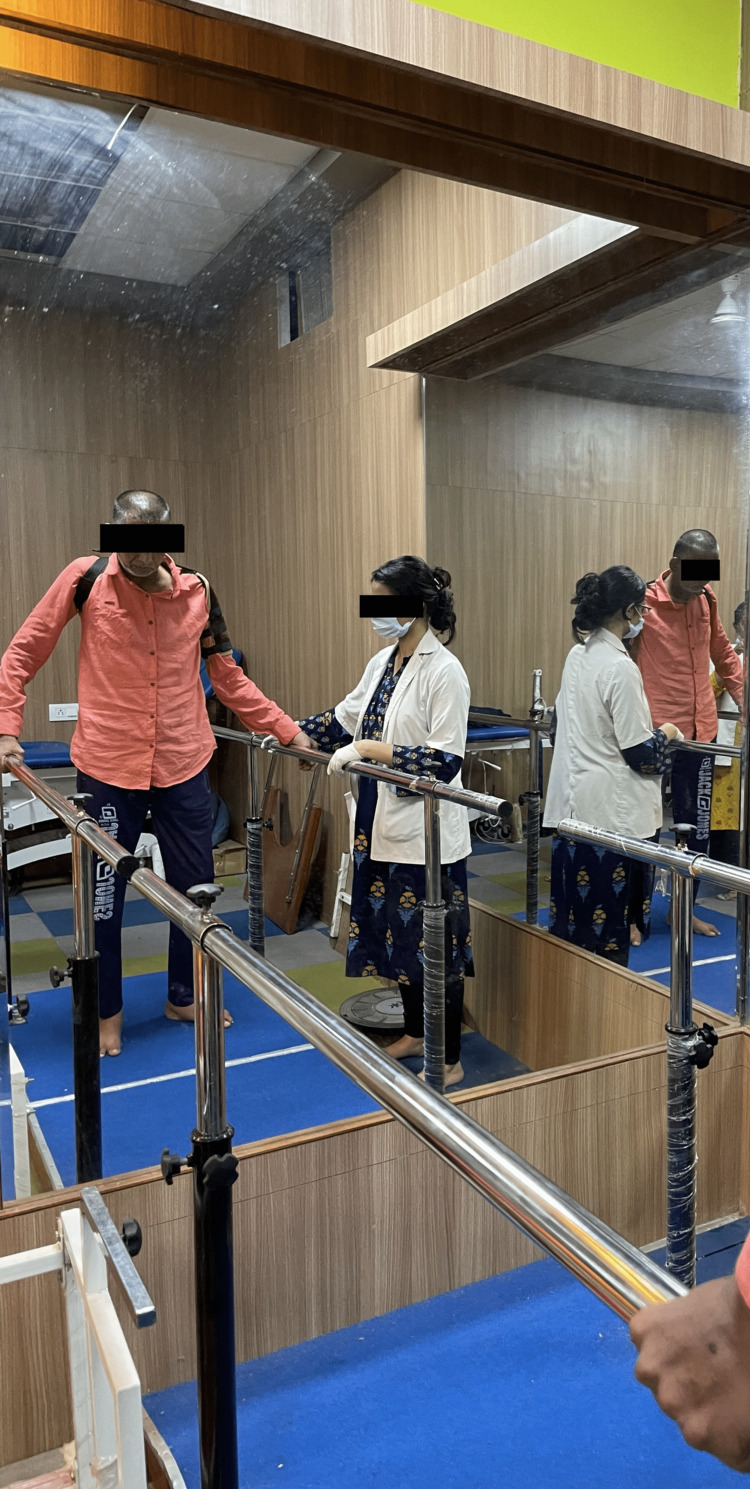
Patient walks inside parallel bars in front of a mirror

The treatment protocol was devised according to the patient's needs and the limitation faced by him. The treatment interventions (Table 4) were specific to the problems that were identified. Figure 3 shows adductor stretching. Figure 4 shows the PNF pattern for the lower limb. Figure 5 shows parallel-bar walking.

**Table 4:** Physiotherapeutic interventions

Problem identified	Probable cause	Goals	Physiotherapeutic Intervention
Tightness and disuse of the right upper limb	Prolonged immobility and inactivity	To preserve the properties of musculoskeletal structures, maintain joint integrity and mobility	Passive movements: shoulder flexion/extension, abduction-adduction, Internal-external rotation, (10 repititions [reps] performed for 1 set). Self-assisted arm movements: lifting the affected arm with the help of the non-affected arm, have the patient clasp his hand together, fingers crossed together, (10 reps for 1 set). Table-top polishing: the patient sits in front of a table/platform (can be done even when the patient is seated with his back supported on the bed) with protracted scapula, elbow in extension, and both hands positioned on a towel. With the unaffected arm, the patient pulls on the towel sideways and forward, moving the unaffected arm, (10 reps for 1 set). Achilles tendon stretch bilaterally, (10 times for 1 set). Adductor stretch to both the legs, (10 times for 1 set). Adductor stretch is depicted in Figure 3.
Spasticity in shoulder flexors and elbow flexors	Spasticity	To normalize the muscle tone	Rood's inhibitory approach (deep tendinous pressure, prolonged icing for 20 minutes, sustained stretching for three times with a hold of 60 sec).This method was developed by Margaret S. Rood [9].
Generalized weakness of the unaffected limbs	Post-stroke weakness	To strengthen the unaffected side upper limb and lower limb	Progressive resisted exercises. Use a 1kg weight cuff for ten repetitions and then gradually increase the weight.
Inability to perform movements of lower limb	To improve neuromuscular control and function.	To enhance the recovery of the right lower limb to achieve normal biomechanics of lower limb.	Proprioceptive neuromuscular facilitation (PNF) patterns for the lower limb. The rhythmic initiation technique is used, in which the patient is first treated with active-assisted movement with the help of verbal cues taught the missing component, and the pattern of movement is corrected. Then, resistance is applied as the patient can perform the corrected actions. Diagonal 1 (D1):flexion-extension; D1 Fexion (D1 Flx): Hip is flexed-adducted-externally rotated, knee flexed, ankle dorsiflexed and brought into inversion. D1 Extension (D1 Ext): Hip is extended-abducted-internally rotated, knee flexed, ankle plantarflexed and brought into eversion. Diagonal 2 (D2): Flexion-extension; (D2 Flx): Hip is flexed, abducted, internally rotated, knee flexed, ankle dorsiflexed and brought into eversion. (D2 Ext): Hip is extended, adducted, and externally rotated. The PNF for lower limb is depicted in Figure 4.
Difficulty walking independently	Gait-abnormalities like pelvic retraction and elevation, hip adduction and internal rotation. Preliminary swing phase leading to decreased ground clearance. Inadequate dorsiflexion, leading to the absence of heel-strike phase.	To achieve correct posture and relearn walking	Walking in front of a mirror: Parallel-bar walking. The patient holds the railings, and he is taught, Toe-off or dorsiflexion of right forefoot ten times. Placing weight on the disabled lower leg. Walking in front of a mirror with verbal cues to increase the right leg swing. Walking in front of mirror (Parallel Bar) is depicted in figure 5.
Difficulty in stair-climbing.	Inadequate hip and knee flexion	Achieving hip and knee flexion	Walking in front of a mirror, parallel-bar walking with a stepper placed in front of the patient, the patient was instructed to climb up and down the stepper. This exercise is done ten times.
Facial palsy	Upper motor-neuron Lesion.	To strengthen the muscles of the face.	Facial muscles PNF. Home exercise program for facial muscles with mirror biofeedback.

Follow-up and outcome measures

There was a significant improvement in the patient's motor functions and activity of daily living. There was a ten-point increase in the Motor Assessment Scale after the rehabilitation. The strength of the facial muscles also showed improvement after the facial exercise program as there was a twenty-nine-point increase in the Sunnybrook Facial Grading System. There was an eight-point improvement in the Dynamic Gait Index; this suggested that the patient's balance had improved, and all treatment strategies contributed to his progress and recovery from his impairment. After six weeks of integrative neuro-physiotherapy approaches, there were notable improvements in the patient’s condition; the comparison between the pre-treatment and post-treatment scores is depicted in Table 5.

**Table 5:** The pre-treatment and post-treatment outcome measures after six weeks of integrative neurophysiotherapy approaches

Outcome Measure	Pre-Treatment score	Post-Treatment score
Motor Assessment Scale	14	24
Dynamic Gait Index	12	20
Sunnybrook Facial Grading Scale	58	87

## Discussion

 A stroke can be defined as a sudden onset of focal neurologic deficit due to a hemorrhage or ischemia [10]. The most frequently occurring stroke is the MCA stroke. MCA supplies the whole lateral cerebral hemisphere. If major territories are involved, this stroke can lead to severe impairments in the patient for the rest of his life. Contralateral hemiparesis, hemisensory loss of two-thirds of the face and upper limb, and aphasia are some of the severe disabilities faced due to an MCA occlusion stroke. The function of the lower extremities is more spared than that of the facio-brachial area [11]. Diagnosis of stroke is confirmed with CT and MRI [12]. MRI is done to identify the affected area in greater detail. The medical treatment usually revolves around thrombolysis, thrombectomy, and administering anticoagulant and antiplatelet medications. At the same time, physical therapy employs various treatment modalities and methods to deal with the physical limitations following a stroke. It has been proven that physical therapy rehabilitation is effective in addressing and improving the patient's motor impairments [13]. Facial palsy can be treated with facial PNF [14] and facial exercises [15]. Stroke is a common condition, which occurs around the globe, but with varying levels of involvement of different lobes in each individual case. Each individual case is different. In large randomized control trials, literature reviews, attention to an individual's impairment, and problem-based interventions are not administered. Case reports with classic MCA infarct treated only with PNF for facial palsy and simultaneously for hemiplegia are not present. This case exhibits major involvement of the left frontoparietal-temporo-occipital lobe leading to expressive aphasia, facial palsy, and right-hand paralysis (more than the leg). In this case analysis, the patient showed significant improvement after receiving physical therapy sessions, particularly the PNF, which was administered from an early stage. His motor abilities increased, and he is more likely to be able to perform important activities of daily living by himself.

## Conclusions

The increasing stress levels and the co-morbidities like high blood pressure, diabetes mellitus, and abuse of certain substances like alcohol have contributed to a rise in cases of cerebrovascular event. Stroke is known to be a common condition but accounts for major impairments despite the availability of a pantheon of treatment options. The efficacy of a thoroughly monitored physiotherapy program in enhancing the strength and relearning of motor functions is described in this case analysis. It is quite important to provide a tailor-made rehabilitation program according to the current needs and problems of the patient. Thus, to conclude we would like to emphasize that a complete approach combining traditional therapy and a thoughtful physical therapy program significantly improved the patient's health by reducing symptoms and improving his motor skills, which has improved his quality of life by providing physical independence in activities of daily life.
